# Minimum four-year clinical outcomes after on-table reconstruction technique for Dubberley type III in coronal shear fractures of the capitellum and trochlea: a report of 10 patients

**DOI:** 10.1186/s12891-024-07628-2

**Published:** 2024-07-03

**Authors:** Il-Hyun Koh, Jung Jun Hong, Ho-Jung Kang, Yun-Rak Choi, Ji-Sup Kim

**Affiliations:** 1 Department of Orthopaedic Surgery, Yonsei University College of Medicine, Severance Hospital, Seoul, Republic of Korea; 2 Department of Orthopaedic Surgery, Yonsei Wa Hospital, Incheon, Republic of Korea; 3 Department of Orthopaedic Surgery, Yonsei Sarang Hospital, Seoul, Republic of Korea; 4 Department of Orthopaedic Surgery, College of Medicine, Ewha Womans University Seoul Hospital, 260, Gonghang-daero, Gangseo-gu, Seoul, 07985 Republic of Korea; 5 Yonsei University College of Medicine, Seoul, Republic of Korea

**Keywords:** Coronal shear fracture, Capitellum, Trochlea, On-table reconstruction

## Abstract

**Purpose:**

Comminuted coronal shear fractures of the distal humerus represent rare injuries and are difficult to treat, especially comminuted capitellum and trochlear fractures (Dubberley Type III). The on-table reconstruction technique of comminuted articular fractures may be an option, although it has not been reported in the coronal shear fracture of the distal humerus. The aim of the present case series is to determine the functional and radiological outcomes of on-table reconstructed Dubberley III fractures.

**Methods:**

A retrospective review was conducted of 10 patients with Dubberley type III fractures in coronal shear fractures of the capitellum and trochlea who underwent an ‘on-table’ reconstruction technique between January 2009 and October 2019. All patients were evaluated using the disabilities of the arm, shoulder, and hand (DASH) score, American Shoulder and Elbow Surgeons(ASES) score, Mayo Elbow Score Performance Index (MEPI) score and at least 4 years later.

**Results:**

All cases achieved union. At the final follow-up, the mean range of elbow motion was 11.5°of flexion contracture and 131.9° of further flexion. The mean DASH score was 21.2 (5.7) points (range 13.3–32.5). The mean ASES score was 88.6 ± 7.4 (range, 77 to 100). The mean MEPI score was 87 (10) points (range 70–100). In complication, partial osteonecrosis of capitellum is developed in one patient. One patient had heterotopic ossification without functional impairment.

**Conclusion:**

The on-table reconstruction technique can be a reliable option in the surgical treatment of complex distal humerus fractures. This technique allows anatomical reduction of comminuted capitellum and trochlea, with a low risk of avascular necrosis over 4 years of follow-up.

**Level of evidence:**

Level IV, retrospective case series.

## Introduction

Coronal fractures of the distal humerus, which involve capitellum or trochlea fractures are challenging injuries, and their treatment options are diverse and complex. Among these, Dubberley type III, comminuted separate capitellum and trochlea fractures, typically have a worse prognosis due to its articular comminution [1]. An increase in articular comminution is linked to poorer outcomes, primarily due to the technical challenges of achieving anatomical reduction and stable fixation.

The appropriate method for treating Dubberley type III fractures is still controversial. Surgical treatment options include open reduction and internal fixation (ORIF) [2, 3], fragment excision [4, 5], and total elbow arthroplasty(TEA) [6, 7].

ORIF could be the best option if the fracture is reduced anatomically. Yet, because of the comminution and poor vision, achieving anatomical reduction of this articular surface is difficult. In addition, avascular necrosis (AVN) [3, 8, 9] and fixation failure [1, 10] can occur even after the proper bony reduction.

Excision of the fragments that involve the trochlea may be associated with poor outcomes because of secondary arthritis, pain, and stiffness [5]. Primary TEA could be a better option for older patients with these fracture patterns [11]. Short-term functional outcomes following TEA for distal humeral fractures tend to be good, but long-term complications, such as loosening and peri-prosthetic fractures, could be challenging to treat. Additionally, in relatively young active patients, it is difficult to consider primary elbow arthroplasty in terms of long-term durability.

Currently, there are no report in the literature about the extra-corporeal ‘on-table’ reconstruction of Dubberley type III fractures. Therefore, we conducted a retrospective study to evaluate the clinical and radiological outcomes of patients with comminuted capitellum and trochlear fractures (Dubberley Type III) that were treated with open reduction with an on-table reconstruction technique and internal fixation. The primary aim of this study was to examine the rate of bony union during the follow-up period. The secondary aims of the study were to examine the mid-term clinical outcomes and to identify the occurrence of any complications. We hypothesize that this technique leads to good functional results and bony union with minimal postoperative complication.

## Methods

The study design and data collection were approved by the institutional review board of our hospital’s Human Experimental and Ethics Committee (IRB No. 9-2022-0019), and informed consent was obtained from all participants. We retrospectively reviewed 32 consecutive patients who had undergone open reduction and internal fixation for coronal shear fractures of the capitellum and trochlea fracture between January 2009 and October 2019. Included in the study were patients (1) who underwent on-table reconstruction of Dubberley Type III fractures (2) who had at least 4 years of clinical follow-up data. Fractures were classified according to Dubberley’s classification: a fracture of the capitellum with or without the lateral trochlear ridge (type 1), fracture involving the capitellum and the trochlea as one single piece (type 2) or as separate fragments (type 3). Absence (A) or presence (B), of posterior comminution was also assessed [1]. Patients were excluded for the following reasons: (1) ipsilateral concomitant fracture of elbow and wrist, including radial head fracture, (2) previous injuries of the affected limb. The diagnosis was confirmed by radiographic assessment, including anteroposterior and lateral views and both-oblique views of the elbow for all patients. CT scan to analyze the fracture and plan the operative treatment. The demographic data are shown in Table 1.

**Table 1:** Demographics

No	Sex	Age	Injured side	lateral condyle fracture
1	M	52	R	0
2	F	78	L	+
3	F	55	R	+
4	M	38	R	0
5	F	62	L	0
6	F	49	R	0
7	F	52	L	+
8	M	40	L	0
9	F	40	R	0
10	F	61	R	0

M male, F female, R right, L left

The patients were evaluated by two independent orthopaedic surgeons, who were not involved in the surgery and patient treatment, using an array of tools. All follow-up data were collected through in-person visits. In case of loss to follow-up, we contacted the individuals by phone and encouraged them to visit. The following were applied as functional indices: pain (10-point visual analogue scales), active range of motion, Disabilities of the Arm, Shoulder, and Hand (DASH) score [12], the American Shoulder and Elbow Surgeons score [13] and the Mayo Elbow Performance Index (MEPI) score [14]. The MEPI consists of four parts: pain (with a maximum score of 45 points), ulnohumeral motion (20 points), stability (10 points) and the ability to perform five functional tasks (25 points). The total score ranges from 5 to 100 points, with higher scores indicating better function. If the total score is included between 90 and 100 points, it can be considered excellent; between 75 and 89 points, good; between 60 and 74 points, fair; and less than 60 points, poor [15].

The radiographic examinations were used to detect non-unions, inadequacy or loss of reduction, AVN, heterotopic ossifications (according to the Hastings and Graham system) and signs of posttraumatic arthritis (according to the Broberg and Morrey classification). Each patient was assessed for operation-related complications (nerve injury, infection).

### Surgical technique and postoperative rehabilitation

**Fig. 1:**
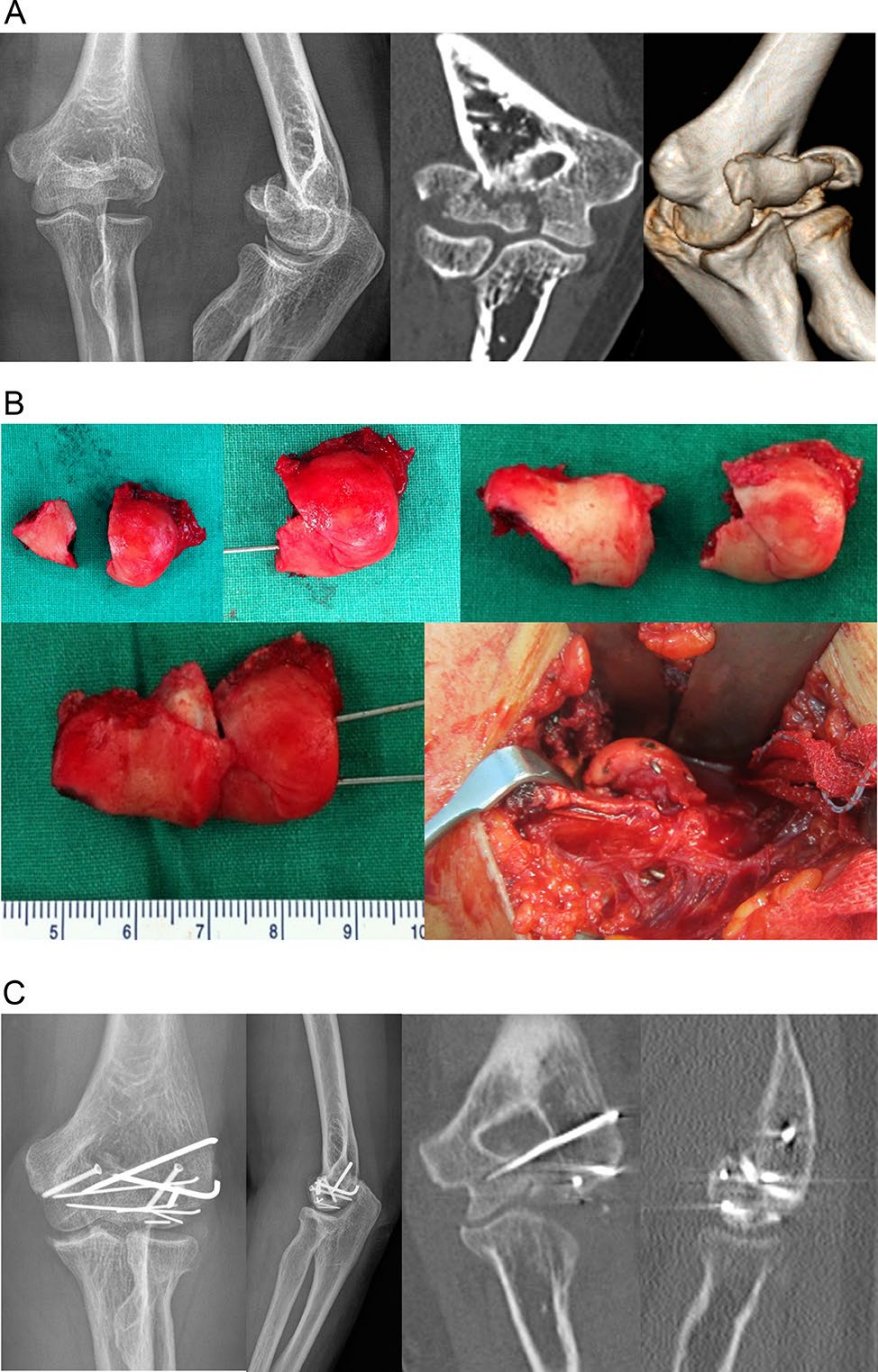
(**A**) Preoperative x-ray and CT scans of Dubberley type IIIA fracture. (**B**) ‘on-table’ reconstruction using subchondral K-wires. (**C**) postoperative x-ray and CT scan showing union after 1 year

A single attending surgeon (H.J.K.) with expertise in treating distal humerus fracture, Level 4 [16], performed all operations. The patient was placed in the supine position, and the affected arm was draped in a sterile manner to allow free manipulation. The distal humerus fracture site was exposed using the anterolateral approach. In casese of Dubberley type 3 fractures, ‘on-table’ technique was used (Fig. 1). The separated capitellum and trochlea fracture fragments were retrieved from the joint, and subchondral K wire fixation of separated capitellum and trochlea fragments was done extracorporeally. The reduced articular fragments were repositioned and fixed to the distal humerus using mini-screws or headless compression screws (Medartis, Basel, Switzerland), anterior to posterior direction. The lateral collateral ligament, when injured, was reattached to its humeral origin with suture anchor sutures. When a final fixation was achieved, elbow range of motion and stability were tested. After surgery, a long arm splint was applied for 4 weeks. After splint removal, patients began a hand physical therapy program for 8 weeks, including hand and wrist edema control using active elbow motion exercises. The patients were followed up every 2 weeks until 3 months and by 8-week intervals afterward.

### Statistical analysis

All continuous variables were expressed as mean with standard deviation (SD) or median with an interquartile range after testing for normality using the Shapiro–Wilk test. All discrete variables were expressed as a frequency or ratio.

## Results

**Fig. 2:**
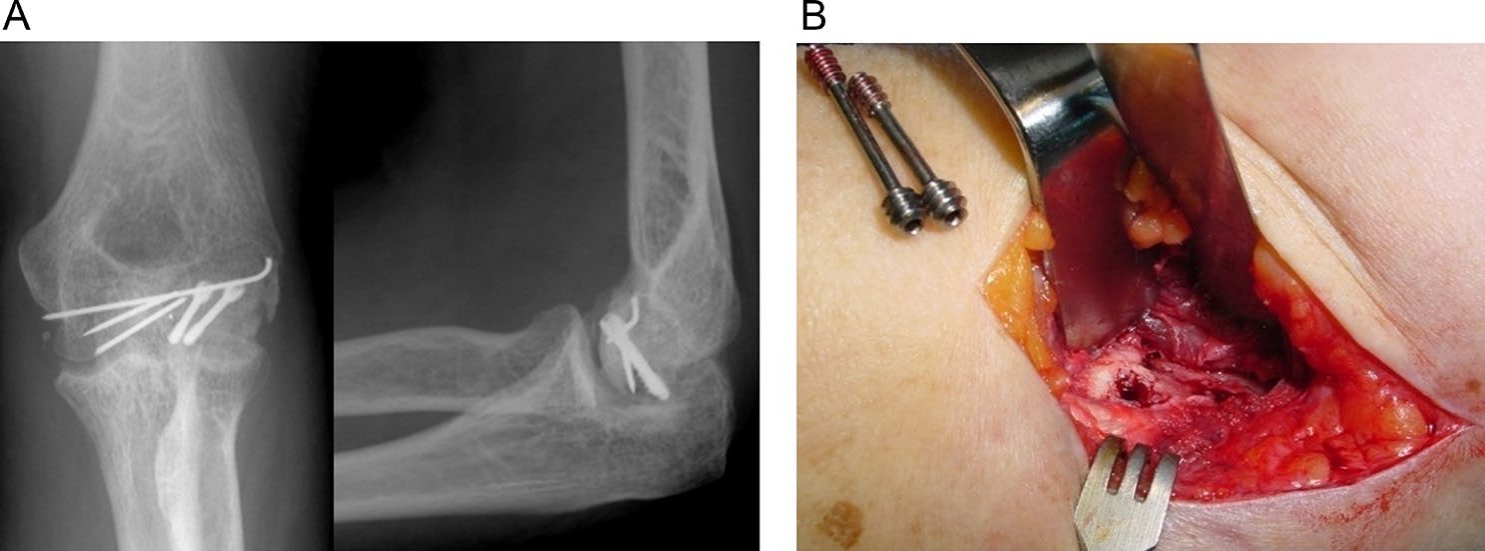
(**A**) x-ray showing partial necrosis of capitellum at 3months postoperatively. (**B**) hardware removal and arthrolysis at 1 year postoperatively

After assessing all patient records, 10 patients with Dubberley Type 3 fractures, which fulfilled our criteria, were included in this study. The patient’s demographic data and preoperative clinical status are described in Table 1. Of those, 3 patients had concomitant lateral condyle fracture. These injuries were further classified by the absence (A) or presence (B) of posterior comminution according to Dubberley classification [1], and all fractures were type 3 A. Three patients had a complete lateral ligamentous injury that was identified intra-operatively. The mean age of patients was 52.7 ± 5.7 years (range: 38–78 years). 7 patients were female, and 3 were male. 6 fractures occurred in the dominant hand. The mean follow-up period was 54.7 ± 4.8 months (range: 48–60 months). At the last visit, the mean American Shoulder and Elbow Surgeons score was 88.6 ± 7.4 (range, 77 to 100). The mean DASH score was 21.2 ± 5.7 (range: 13.3–32.5). The mean Mayo Elbow Performance Score was 87 ± 10 points (range: 70–100 points), with 5 excellent, 4 good, and 1 fair. The VAS was 2 ± 1 (range: 0–4). The active range of motion (flexion–extension arc) was 119.9° ± 9.9° (range: 105°–133°), with a mean flexion of 132° (range: 125°–136.5°) and a loss of extension of 11.5° (range: 5°–25°). No prono-supination limitation occurred in any of the patients included in the analysis. The postoperative CT scan revealed anatomical articular reduction in all patients. There was no loss of reduction during follow-up. Fracture union was obtained in all patients at a mean of 10.8 ± 3.2 weeks (range, 10–20 weeks). During the follow-up period, one patient developed asymptomatic partial AVN of the capitulum at 3months postoperatively (Fig. 2). We performed screw removal and arthrolysis 1-year postoperatively, and AVN did not progress afterwards. At the final visit, this patient had a Mayo Elbow Performance Score of 70 points (fair result) and the VAS was 3 (Table 2, patient number 7). Another patient developed heterotopic ossification developed without functional impairment (Class I in Hastings and Graham classification).

**Table 2:** Results

No	DASH	ASES	MEPI	ROM extension (°)	ROM Flexion (°)	Flexion – extension arc (°)	ROM Pronation (°)	ROM Supination (°)	Return to work (weeks)	additional operation	AVN	Follow-up (months)
1	25.8	85	85	5	135	130	90	80	10	0	0	60
2	13.3	95	85	10	125	115	90	90	8	0	0	56
3	19.2	100	100	5	140	135	90	90	8	0	0	50
4	17.5	92	90	0	135	135	90	80	8	0	0	48
5	18.3	97	100	5	141.5	136.5	90	85	8	0	0	60
6	20	82	85	10	140	130	90	90	9	0	0	55
7	20.8	85	70	20	135	115	90	90	12	+	+	50
8	15.8	93	100	15	140	125	90	90	8	0	0	58
9	32.5	77	75	25	135	110	90	90	15	+	0	60
10	29.1	80	80	20	130	110	90	90	12	+	0	50

DASH Disabilities of the arm, shoulder, and hand score, ASES American shoulder and elbow surgeon score, MEPI Mayo elbow performance index score, ROM range of motion, AVN avascular necrosis

## Discussion

This is the first report demonstrating the functional and radiological outcomes of the described ‘on-table’ reconstruction technique for treating Dubberley type 3 fractures. All fractures showed bony union with good functional outcome at the follow-up’s end, although one partial AVN occurred, constituting 10% of our cases.

The ‘on-table’ reconstruction technique for comminuted articular fractures was initially introduced in radial head fractures [17, 18]. A. Businger et al. [17] reported 6 cases of Mason type 3 and type 4 radial head fractures using the ‘on-table’ reconstruction technique. 5 fractures were united, and 1 patient went on to radial head excision due to AVN of the radial head and pain of elbow. However, after the mean 9 years of follow-up, all showed good functional outcomes, the mean DASH score was 1.94 points, and the mean range of movement was 6°–141° at the elbow. The ‘on-table’ reconstruction technique we employed for comminuted articular fractures in the distal humerus also demonstrated satisfactory treatment outcomes similar to those reported in radial head fractures.

AVN after capitellum or trochlear fracture fixation was also reported in other studies. A recent meta-analysis by M. Heller et al. [19] shows 12% of AVN in capitellar fractures. To be more specific with Dubberley type III fractures, Dubberley et al. [1] reported 1 AVN in three patients with Dubberley type IIIA fractures and 2 AVNs in eight Type IIIB patients. Durakbasa et al. [20] reported 4 AVNs out of 7 Dubberley type III patients. Compared to these studies, our result of an AVN rate of 10% is even better.

This relatively low rate of AVN could be stemmed in our limited approach. We use a lateral approach only, thus saving medial side soft tissue. Dubberley et al. [1] and Durakbasa et al. [20] also used a single posterior incision. Still, they dissected medially and laterally through this incision, which could interfere with circulation to the fracture site. Our single-sided approach is possible as we reduce outside the fracture site, not through the medial side.

We acknowledge that on-table reconstruction could harm the biological environment because fragments should be stripped off from the surrounding soft tissue. However, it is known that the blood supply to the capitellum and lateral trochlea comes mainly from the posterior condylar perforating vessels [21]. This means separating the fragment from the anterior side may not harm the blood supply. This hypothesis is also supported by a meta-analysis published in 2023 by M. Heller et al. [19] This study reported that when the screw is inserted at the capitellum from anterior to posterior, the mean AVN rate is 11%, which is lower than the mean AVN rate of 29% when the screw was inserted posterior to anterior.

On top of that, there is insufficient evidence that this AVN could lead to poor clinical outcomes. S. Mukohara et al. [22] suggested that AVN may not be important, but whether the trochlear component can be reconstructed may be important. They reported that patients with three or more fragments of trochlea had worse clinical outcomes and ROM than those with fewer trochlear fragments. Our patient with AVN also showed fair results afterwards. From this point of view, we used our technique to reconstruct the trochlear component correctly.

## Limitations

This study had several limitations. The value of our study may be limited by the small number of cases and the retrospective analyses of the data, which is susceptible to selection bias. Nevertheless, Dubberley type III, capitellum and trochlea fractures are rare, and the reported results are important for understanding and optimizing treatment options. Furthermore, there were no posterior comminution (type B) fractures. Our relatively good functional outcome and lower AVN could contribute to fewer extensive cases. However, our fixation method, which inserts screws from the anterior side, is still valid for type B fractures, because we do not further damage posterior side, where main blood supplies presents. Of course, a further study, including more severe cases, is needed to support our theory fully. Lastly, our results were limited to mid-term follow-up; further investigation with long-term follow-up is needed.

## Conclusions

Using an ‘on-table’ reconstruction technique, we have obtained bony union in all cases of Dubberley type III fractures, with good functional results without severe complications and only one case of AVN. Therefore, despite some limitations, we conclude that, with a personal follow-up of a minimum 4 years postoperatively, an ‘on-table’ reconstruction and fixation of a severe comminuted fractures of the capitellum and trochlea may be considered a reliable and safe treatment option. Moreover, we believe that our findings can provide some clues to future studies regarding the importance of the anatomical reduction of the articular surface over preserving soft tissue attachments around fracture site in achieving good outcomes.

## Data Availability

Availability of data and materials: The datasets used and/or analysed during the current study available from the corresponding author on reasonable request.
